# Supplementary material for: The inability to walk unassisted at hospital admission as a valuable triage tool to predict hospital mortality in Rwandese patients with suspected infection
Source: PLoS One. 2020 Feb 21;15(2):e0228966. doi: 10.1371/journal.pone.0228966 (PMC7034857; doi:10.1371/journal.pone.0228966)
Supplement: Attachment — Submitted filename: Papertext.docx [file pone.0228966.s002.docx]

**The ability to walk at hospital admission as a valuable triage tool to predict disease severity and outcome in Rwandese patients with suspected infection**

**Short title: Ability to walk as a predictor of disease severity in infection**

Arthur Kwizera, MD^1^; Olivier Urayeneza, MD, FACS^2,3^; Pierre Mujyarugamba, MSc^2^; Jens Meier, MD^4^; Andrew J. Patterson, MD, PhD^5^; Lori Harmon, RRT, MBA^6^; Joseph C. Farmer, MD, FCCM^7^; Martin W. Dünser, MD^4^; for the “Sepsis in Resource-Limited Nations” Task Force of the Surviving Sepsis Campaign

^1^, Department of Anaesthesia and Critical Care, Makerere University College of Health Sciences, Kampala, Uganda; ^2^, Gitwe Hospital and Gitwe School of Medicine, Gitwe, Rwanda; ^3^, Department of Surgery, California Medical Center, Los Angeles, United States of America; ^4^, Department of Anesthesiology and Intensive Care Medicine, Kepler University Hospital and Johannes Kepler University Linz, Linz, Austria; ^5^, Department of Anesthesiology, Emory University, Atlanta, Georgia, United States of America; ^6^, Society of Critical Care Medicine on behalf of the Surviving Sepsis Campaign, Mount Prospect, IL, United States of America; ^7^, Department of Critical Care Medicine, Mayo Clinic, Phoenix, AZ, United States of America.

*Address for Correspondence:*
PD Dr. Martin W. Dünser, DESA, EDIC; Department of Anesthesiology and Intensive Care Medicine, Kepler University Hospital and Johannes Kepler University Linz, Altenbergerstrasse 69, 4040 Linz, Austria; Tel.: ++43 5 7680 83 2158, Email: [Martin.Duenser@kepleruniklinikum.at](mailto:Martin.Duenser@kepleruniklinikum.at)

**ABSTRACT**

**Objective:** To assess the value of the inability to walk unassisted to predict hospital mortality in patients with suspected or proven acute infection in a resource-limited setting.

**Methods:** This is a *post hoc* study of a prospective, before-and-after trial performed in a rural hospital in Rwanda. Patients hospitalized because of a suspected or proven acute infection, were aged ≥2 years and able to walk unassisted before this disease episode were included. At hospital presentation, the walking status was graded into: 1) can walk unassisted, 2) can walk assisted only, 3) cannot walk. Two-by-two tables were used to determine the sensitivity, specificity, negative and positive predictive values of the inability- to- walk- unassisted to predict in-hospital death.

**Results:** One-thousand-sixty-nine patients were included. Two-hundred-one (18.8%), 315 (29.5%), and 553 (51.7%) subjects could walk unassisted, walk assisted or not walk at hospital presentation, respectively. Their hospital mortality was 0%, 3.8% and 6.3%, respectively. The inability to walk unassisted had a low specificity (20%) but was 100% sensitive (CI95%, 90-100%) to predict in-hospital death (p=0.00007). Fifteen (7.5%), thirty-four (10.8%) and one hundred sixty-seven (30.2%) patients who could walk unassisted, walk assisted or not walk at hospital admission presented with a qSOFA score count ≥2 points, respectively (p<0.001). The inability to walk unassisted correlated with a diagnosis of meningitis and urinary tract infection, an altered mental state, respiratory distress, the qSOFA score count, the incidence of pediatric sepsis, heart rate, respiratory rate, body temperature, white cell count and hemoglobin levels, length of hospital stay, and costs of care.
**Conclusions:** Our results suggest that the inability to walk unassisted at hospital admission is a highly sensitive predictor of in-hospital mortality in Rwandese patients with a proven or suspected infection. The walking status at hospital admission appears to be a crude indicator of disease severity.

*Word count abstract:	298 words*

**INTRODUCTION**

Infectious diseases and sepsis are leading causes of death in resource-limited nations [1,2]. Unfortunately, in a resource-limited environment differentiating between patients with significant infection who will develop life-threatening sepsis complications versus patients who will manifest serious infection only is difficult. Furthermore, lack of routine diagnostic technology hampers the identification of patients with the highest risk of developing life-threatening sepsis [1,3]. We aimed to seek an additional assessment methodology that is simple, directly applicable to this patient population, and easy to accomplish and interpret. Longer term, it is our goal to translate improved identification of patients at hospital admission with the highest risk of death from infection into increased clinical support where appropriate.

In a recent prospective, before-and-after feasibility trial, our working group showed that a focused education program and implementation of an infection treatment bundle in clinical practice increased the rate of early evidence-based interventions in patients with acute infections (mostly malaria) admitted to a Sub-Saharan African district hospital [4]. In view of the fact that all patients who were hospitalized with an acute infection were included into this study, the overall disease severity and mortality (3.8%) was low [4]. In order to reduce the death toll from infectious diseases in this setting, future research efforts should focus primarily on patients with an acute infection who are at risk of in-hospital death. While some modified illness scores have shown promise [5,6], no triage tool exists which could early and reliably differentiate between patients with a minimal risk of death and patients who are at risk of dying from their infectious process.

In this analysis, we assessed the value of the inability to walk unassisted at hospital presentation to predict in-hospital mortality in patients with suspected or proven infection hospitalized at the Gitwe District Hospital in rural Rwanda. We hypothesized that this simple prognostic indicator of disease severity accurately portended in-hospital death.

**MATERIALS AND METHODS**

This is a *post hoc* analysis of an investigator-initiated, single-center, prospective, before-and-after feasibility trial conducted at the adult and pediatric emergency department of the Gitwe District Hospital in Rwanda. The trial was conducted between March 2016 and March 2017 [4]. Originally, we tested whether a focused education program and consistent implementation of a simplified severe infection and sepsis treatment bundle increased the rate of early evidence-based interventions (antimicrobial therapy, surgical source control, blood glucose measurement and, in cases of hypoglycemia IV glucose administration, recovery position in cases of unresponsiveness to touch and/or pain, oxygen administration in cases of respiratory distress, fluid administration in cases of systemic hypoperfusion). The study population consisted of 1,594 patients aged >28 days of life who were hospitalized because of a proven or suspected acute infection. Exclusion criteria were refusal to provide written informed consent. The trial protocol was pre-published (www.clinicaltrials.gov; NCT02697513), reviewed and approved by the National Health and Research Committee as well as the Rwanda National Ethics Committee (No.007/RNEC/2016). Written informed consent was obtained from all study patients or parents/next of kin in children.

*Setting*

Details about the study site were previously published [4]. Briefly, the Gitwe District Hospital serves a population of approximately 160,000, has 200 beds and one emergency department with a separate adult and a pediatric admission room. No intensive care or high dependency unit is available, and supporting facilities such as the laboratory or radiology department are only modestly equipped. Patients usually reach the hospital by foot, private cars, or when referred from regional healthcare centers ,by the hospital’s own ambulance.

*Study Patients*

For this *post hoc* analysis, we included only study patients who were able to walk before they sought care for this specific disease episode. In children aged two years or older, this information was obtained from the parent, next of kin, or legal representative accompanying the child.

*Data Extraction*

In the original trial, the walking status was evaluated at hospital admission by recording how the patient entered the emergency department. The patient’s ability to walk was graded into three categories: 1) can walk unassisted, 2) can walk only assisted, 3) cannot walk. This information was extracted from the trial database for all study patients fulfilling the inclusion criteria for this *post hoc* analysis. In addition, the following variables were extracted: patient demographics, HIV status, pre-existent malnutrition, presumed infection source, danger signs (altered mental state, respiratory distress, systemic hypoperfusion), vital parameters (heart rate, respiratory rate, systolic blood pressure, capillary refill time, temperature), laboratory results (blood sugar, white cell count, hemoglobin levels whenever determined) and the quick Sequential Organ Failure Assessment (qSOFA) score count [7] at hospital admission. The length of hospital stay, total costs of care and survival status at hospital discharge were also extracted.

*Study Endpoints and Statistical Analysis*

The primary endpoint of this analysis was to determine the value of the inability to walk unassisted at hospital admission as a predictor of in-hospital mortality. Secondary outcomes were the relationship between the walking status at hospital admission and the incidence of sepsis, the source of infection, the presence of danger signs, vital parameters, the qSOFA score count, length of hospital stay as well as total costs of care. The qSOFA score (≥2 points) was used to identify adult patients with an increased risk of death (*i.e.* sepsis) as suggested by the Sepsis3 study group [7]. In children (<15 years), an increased risk of death (*i.e.* sepsis) was assumed if the child had a suspected or proven infection with any one of the following criteria [8]: body temperature <36°C, body temperature >38°C plus altered mental status, body temperature >38°C plus respiratory distress, body temperature >38°C plus a history of not feeding, or body temperature >38°C plus convulsions.

The statistical analysis was performed using the PASW statistical software package (IBM SPSS Statistics 20; IBM, Vienna, Austria). Two-by-two tables were used to determine the sensitivity, specificity, as well as the negative and positive predictive values of the inability to walk unassisted at hospital admission to predict in-hospital death. For this analysis, we grouped patients into subjects who could walk unassisted and those who could not (thus combining the categories can walk assisted only and cannot walk). Descriptive methods were used to evaluate in-hospital mortality of patients with a qSOFA score count ≥2 points as categorized by their walking status at hospital admission. The Chi squared test and an analysis of variance were used to compare binary and linear variables between the three categories of walking status at hospital admission, as appropriate. In view of the fact that 25 comparisons were performed between the three categories, we adjusted the *p*-value for the number of comparisons (Bonferroni correction) and considered *p*<0.002 to indicate statistical significance. All data are given as median values with interquartile ranges, if not otherwise stated.

**RESULTS**

Of 1,594 patients enrolled into the original study, 1,069 study patients were included into this *post hoc* analysis (Table 1). Reasons for excluding 525 subjects were: age <2 years (n=359)­­, inability to walk unassisted despite of an age ≥2 years (n=141), and missing documentation of the walking status (n=25). Two-hundred-one (18.8%), 315 (29.5%), and 553 (51.7%) study patients could walk unassisted, walk assisted or not walk at hospital presentation, respectively. The inability to walk unassisted at hospital admission had a low specificity but was 100% sensitive to predict in-hospital mortality (p=0.00007) (Figure 1). Vice versa, the ability to walk unassisted at hospital admission had a 100% specificity but low sensitivity to predict hospital survival.

**Table 1. Clinical and demographic features of 1,069 patients admitted to hospital with proven or suspected infection.**

| **Variable** | **Unit** | **All patients** |
| --- | --- | --- |
| *N* |  | 1,069 |
| **Age** | *years* | 20 (7-40) |
| **Male gender** | *n (%)* | 486 (45.5) |
| **HIV positive** | *n (%)* | 33 (3.1) |
| **Malnutrition** | *n (%)* | 7 (0.7) |
| **Infectious focus** |  |  |
| Meninges | *n (%)* | 18 (1.7) |
| Respiratory | *n (%)* | 131 (12.3) |
| Abdominal | *n (%)* | 13 (1.2) |
| Puerperal | *n (%)* | 11 (1) |
| Gastrointestinal | *n (%)* | 81 (7.6) |
| Urinary tract | *n (%)* | 49 (4.6) |
| Skin/soft tissue | *n (%)* | 21 (2) |
| Malaria* | *n (%)* | 610 (57.1) |
| Other | *n (%)* | 196 (18.3) |
| **Length of hospital stay** | *days* | 3 (2-5) |
| **Hospital Mortality** | *n (%)* | 47 (4.4) |

*, 61 (5.7%) patients had a co-infection of malaria and another infectious disease.

Data are given as median values with interquartile range, if not otherwise indicated.

**Figure 1.** Hospital survival in patients with proven or suspected infection categorized by their ability to walk.

PPV, positive predictive value; NPV, negative predictive value; *, Chi² test.

Fifteen (7.5%), thirty-four (10.8%) and one hundred sixty-seven (30.2%) study patients who could walk unassisted, walk assisted or not walk at hospital admission had a qSOFA score count ≥2 points at hospital admission (Figure 2A), respectively. Hospital mortality differed between these patients when they were categorized by their walking status but this did not reach statistical significance (Figure 2B).

**Figure 2.** Incidence of a qSOFA score count ≥2 points (indicating sepsis) at hospital admission (A) and sepsis-related in-hospital mortality (B) in patients categorized by their ability to walk at hospital admission.

*, Chi² test.

The walking status at hospital admission correlated with the diagnoses of meningitis and urinary tract infection, an altered mental state, respiratory distress, the qSOFA count, the incidence of pediatric sepsis, heart rate, respiratory rate, body temperature, white cell count and hemoglobin levels at hospital admission, as well as length of hospital stay, costs of care and survival status at hospital discharge (Table 2).

**Table 2.** Relationships between the ability to walk at hospital admission and infection source, danger signs, vital parameters, and laboratory values at hospital admission as well as outcome parameters and costs of care.

| **Variable** | **Unit** | **Can walk unassisted** | **Can walk assisted** | **Cannot walk** | **Spear-man Rho** | ***p*-value** | |
| --- | --- | --- | --- | --- | --- | --- | --- |
|  | | 201 | 315 | 553 |  | |  |
| **Infectious focus** | | | | | | | |
| **Meninges** | *n* (%) | 0 | 2 (0.6) | 16 (2.9) | 0.1 | 0.001* | |
| **Respiratory** | *n* (%) | 16 (8) | 39 (12.4) | 76 (13.7) | 0.06 | 0.05 | |
| **Abdominal** | *n* (%) | 0 | 5 (1.6) | 8 (1.4) | 0.04 | 0.24 | |
| **Puerperal** | *n* (%) | 4 (2) | 6 (1.9) | 1 (0.2) | -0.08 | 0.006 | |
| **Gastrointestinal** | *n* (%) | 23 (11.4) | 23 (7.3) | 35 (6.3) | -0.06 | 0.04 | |
| **Urinary tract** | *n* (%) | 18 (9) | 16 (5.1) | 15 (2.7) | -0.11 | <0.001* | |
| **Skin/soft tissue** | *n* (%) | 7 (3.5) | 6 (1.9) | 8 (1.4) | -0.05 | 0.11 | |
| **Malaria** | *n* (%) | 105 (52.2) | 179 (56.8) | 326 (59) | 0.05 | 0.12 | |
| **Other** | *n* (%) | 28 (13.9) | 39 (12.4) | 68 (12.3) | -0.02 | 0.43 | |
| **Danger signs at hospital admission** | | | | | | | |
| **AMS** | *n* (%) | 3 (1.5) | 14 (4.4) | 171 (30.9) | 0.35 | <0.001* | |
| **Resp. distress** | *n* (%) | 8 (4) | 20 (6.3) | 77 (13.9) | 0.14 | <0.001* | |
| **Syst. hypoperf.** | *n* (%) | 37 (18.4) | 69 (21.9) | 84 (15.2) | -0.06 | 0.06 | |
| **qSOFA score** | points | 0 (0-1) | 0 (0-1) | 1 (0-1.3) | 0.29 | <0.001* | |
| **Pediatr. Sepsis** | *n* (%) | 7 (3.5) | 19 (6) | 112 (20.3) | 0.15 | 0.001* | |
| **Vital parameters at hospital admission** | | | | | | | |
| **Heart rate** | bpm | 95 (80-112) | 100 (84-112) | 104 (88-117) | 0.13 | <0.001* | |
| **Resp. rate** | bpm | 20 (19-22) | 20 (20-22) | 22 (20-26) | 0.31 | <0.001* | |
| **SBP** | mmHg | 112 (99-122) | 108 (98-121) | 107 (94-121) | -0.08 | 0.05 | |
| **Cap. refill time** | sec | 2 (2-2) | 2 (2-3) | 2 (2-3) | 0.05 | 0.11 | |
| **Temperature** | °C | 37 (36.5-37.9) | 37.1 (36.6-38.2) | 37.3 (36.7-38.5) | 0.12 | <0.001* | |
| **Laboratory values at hospital admission** | | | | | | | |
| **Blood sugar** | mg/dL | 123 (93-161) | 123 (90-156) | 130 (101-166) | 0.11 | 0.12 | |
| **WCC** | G/L | 6.1 (4.6-8.7) | 6.1 (4.1-8.6) | 7.8 (5.3-10.8) | 0.17 | <0.001* | |
| **Hemoglobin** | g/dL | 12.3 (10.6-13.8) | 12.2 (10.1-14) | 11.6 (9.8-13) | -0.13 | <0.001* | |
| **Hospital outcome** | | | | | | | |
| **Length of stay** | days | 3 (2-5) | 3 (2-6) | 4 (2-6) | 0.11 | 0.001* | |
| **Costs of care** | 1,000 RWF | 23.7 (16.2-33.1) | 30.2 (16.9-49.9) | 30.8 (18.6-48.3) | 0.11 | <0.001* | |
| **Survival** | *n* (%) | 201 (100) | 303 (96.2) | 518 (93.7) | 0.11 | <0.001* | |

AMS, altered mental state; Cap. refill time, capillary refill time; Pediatr., pediatric; qSOFA, quick Sequential Organ Failure Assessment; Resp., respiratory; RWF, Rwandan Francs; SBP, systolic blood pressure; Syst. hypoperf., systemic hypoperfusion; WCC, white cell count.

**DISCUSSION**

Rapid identification of patients with serious infection at hospital admission who have the highest risk of death is vitally important if we are to improve their outcome. In a resource limited setting, existing methodologies are not feasible (e.g. measurement of procalcitonin). We believe that there is a need for a simple, binary (Y/N) assessment technique that informs bedside care providers that a patient with serious infection has an increased risk of death from sepsis.

The results of this *post hoc* analysis suggest that the inability to walk unassisted at hospital was a highly sensitive predictor of in-hospital death in Rwandese patients with a proven or suspected infection. When interpreting this finding, it is important to note that over seventy-five percent [4] of the study population received evidence-based interventions for the management of their underlying infectious disease. These evidence-based interventions included: initiation of antimicrobial therapy; surgical source control; blood glucose measurement and intravenous glucose administration (in cases of altered mental state and hypoglycemia, respectively); placement of patient in recovery position (in cases of unresponsiveness to touch and/or pain); oxygen administration (in cases of respiratory distress); fluid administration (in cases of systemic hypoperfusion) [4]. In our cohort, any further intervention to reduce mortality would have only been meaningful in patients who could not walk unassisted at hospital admission. Therefore, future research efforts to test advanced interventions to reduce mortality in patients with an acute infection in a resource-limited setting should focus on subjects who cannot walk unassisted at hospital admission.

It is highly likely that certain confounding factors other than the infectious disease process (e.g. age, comorbidities) influenced the walking status at hospital admission in our patient population. Indeed, both age and comorbidities have been shown to be significantly associated with mortality in patients with severe infection and sepsis in resource-limited settings [9,10]. However, the aim of our study was not to evaluate the independent association between the (in)ability to walk and hospital survival status, but to identify an easy and reliable triage tool with a high sensitivity to predict in-hospital mortality. Therefore, we chose an unadjusted model to determine the crude value of the walking status to predict in-hospital death.

An interesting finding of our analysis was that the walking status at hospital admission was related to the incidence of a qSOFA score count ≥2 points. The qSOFA score has been shown to be a significant risk factor for sepsis and death both in high- [7] as well as low- and middle-income countries [11]. Notably, the fifteen study patients of our cohort who could walk unassisted to hospital admission and had a qSOFA score ≥2 points indicating the presence of sepsis all survived. On the other hand, the hospital mortality of study patients with a qSOFA score ≥2 points who could only walk assisted or who could not walk at all was 2.9% and 8.4%, respectively. Although this difference did not reach statistical significance, it is conceivable and biologically plausible that the ability to walk unassisted may be an indicator of the (otherwise difficult to measure) physiologic reserve of a patient suffering from sepsis.
The walking status at hospital admission was also related to several other indicators of disease severity including danger signs, heart rate, respiratory rate and body temperature, white cell count and hemoglobin levels at hospital admission. Except for the relationship between the ability to walk and altered mental status, and respiratory rate, most of the relationships observed were vague with correlation coefficients <0.2. This suggests that the walking status at hospital admission is a non-specific indicator of the patient’s overall functional condition.

In concordance with our findings, a prospective cohort study from Tanzania reported a clear association between walking status and mortality among medical patients presenting to two hospitals. The ability to walk unaided into the consultation room had a negative predictive value of 97% for hospital mortality [12]. Similarly, the World Health Organization included inability to walk unaided as one of four danger signs to facilitate rapid initiation of empiric anti-tuberculosis treatment among HIV-positive adults with cough [13].

Certain limitations need to be considered when interpreting the results of our analysis. First, we performed multiple comparisons by testing the correlation between 25 parameters and the ability to walk at hospital admission. Although we adjusted the level of significance for this number of comparisons to *p*<0.002, we cannot exclude the possibility that some relationships were chance findings or reflect collinearity. Second, extrapolation of our results to children who cannot walk unassisted or those with premorbid conditions is not possible. Furthermore, extrapolation to other healthcare facilities, other low-/middle-income countries or even high-income countries may neither be possible as the epidemiology of infection (e.g. malaria), modes of transport and standards of care may differ.

**CONCLUSIONS**

Our results suggest that the inability to walk unassisted at hospital admission is a highly sensitive predictor of in-hospital mortality in Rwandese patients with a proven or suspected infection. The walking status at hospital admission appears to be a crude indicator of disease severity.

**ACKNOWLEDGEMENTS**

None.

**REFERENCES**

1. Schultz MJ, Dünser MW, Dondorp AM, Adhikari NK, Iyer S, Kwizera A, et al. Current challenges in the management of sepsis in ICUs in resource-poor settings and suggestions for the future. Intensive Care Med 2017;43: 612-624.
2. Global Burden of Disease Profile: Rwanda. Available from: http://www.healthdata.org/sites/default/files/files/country_profiles/GBD/ihme_gbd_country_report_rwanda.pdf. Accessed June 2018.
3. Rudd KE, Kissoon N, Limmathurotsakul D, Bory S, Mutahunga B, Seymour CW, et al. The global burden of sepsis: barriers and potential solutions. Crit Care 2018;22: 232.
4. Urayeneza O, Mujyarugamba P, Rukemba Z, Nyiringabo V, Ntihinyurwa P, Baelani JI, et al. Increasing evidence-based interventions in patients with acute infections in a resource-limited setting: A before-and-after feasibility trial in Gitwe, Rwanda. Intensive Care Med 2018;44: 1436-1446.
5. Sendagire C, Lipnick MS, Kizito S, Kruisselbrink R, Obua D, Ejoku J, et al. Feasibility of the modified sequential organ function assessment score in a resource-constrained setting: a prospective observational study. BMC Anesthesiology 2017;17: 12.
6. Kruisselbrink R, Kwizera A, Crowther M, Fox-Robichaud A, O'Shea T, Nakibuuka J, et al. Modified Early Warning Score (MEWS) identifies critical illness among ward patients in a resource restricted setting in Kampala, Uganda: A prospective observational study. PLoS One 2016;11: e0151408.
7. Seymour CW, Liu VX, Iwashyna TJ, Brunkhorst FM, Rea TD, Scherag A, et al. Assessment of clinical criteria for sepsis: For the third international consensus definitions for sepsis and septic shock (Sepsis-3). JAMA 2016;315: 762-774.
8. Rahman AE, Iqbal A, Hoque DM, Moinuddin M, Zaman SB, Rahman QS, et al. Managing neonatal and early childhood syndromic sepsis in sub-district hospitals in resource-poor settings: Improvement in quality of care through introduction of a package of interventions in rural Bangladesh. PLoS One 2017;12: e017027.
9. Sakr Y, Jaschinski U, Wittebole X, Szakmany T, Lipman J, Ñamendys-Silva SA, et al. Sepsis in intensive care unit patients: Worldwide data from the intensive care over nations audit. Open Forum Infect Dis 2018;5: ofy313.
10. Jacob ST, Moore CC, Banura P, Pinkerton R, Meya D, Opendi P, et al. Severe sepsis in two Ugandan hospitals: a prospective observational study of management and outcomes in a predominantly HIV-1 infected population. PLoS One 2009;4: e7782.
11. Rudd KE, Seymour CW, Aluisio AR, Augustin ME, Bagenda DS, Beane A, et al. Association of the Quick Sequential (Sepsis-Related) Organ Failure Assessment (qSOFA) Score with excess hospital mortality in adults with suspected infection in low- and middle-income countries. JAMA 2018;319: 2202-2211.
12. Rylance J, Baker T, Mushi E, Mashaga D. Use of an early warning score and ability to walk predicts mortality in medical patients admitted to hospitals in Tanzania. Trans R Soc Trop Med Hyg 2009;103: 790-794.
13. WHO. Improving the diagnosis and treatment of smear-negative pulmonary and extra pulmonary tuberculosis among adults and adolescents, recommendations for HIV-prevalent and resource-constrained settings. Geneva: World Health Organization; 2007. p. 379.
