# Supplementary material for: The inability to walk unassisted at hospital admission as a valuable triage tool to predict hospital mortality in Rwandese patients with suspected infection
Source: PLoS One. 2020 Feb 21;15(2):e0228966. doi: 10.1371/journal.pone.0228966 (PMC7034857; doi:10.1371/journal.pone.0228966)
Supplement: Attachment — Submitted filename: Reviewer comments.docx [file pone.0228966.s001.docx]

Reviewer comments

1. This is a post hoc analysis of a bigger study. In the methods the authors should clearly state this is a nested study of a bigger study and clarify their patient population and refrain from reporting the patient population which was not part of this analysis.
2. There are some results of the bigger study in the methods. This is confusing I would strongly recommend the sentence be removed.
3. The authors state they included patients who could walk before they sought care for this specific disease. Nothing has been reported of the time frame between the last time the patient walked and this disease.
4. The criteria for suspected infection for the adults which was used in patients with a qSOFA score should be clearly defined the way they have defined the pediatric group.
5. Complete reporting of the sensitivity, specificity and all c-statistics would be helpful.
6. Table 1 can be rearranged and remove the unit column and add those in the first column after the variable name.
7. The title and the results do not connect completely.
8. The figures 1 and 2 can easily be reported in text.
